# Identifying the most important facilitators of open research data sharing and reuse in Epidemiology: A mixed-methods study

**DOI:** 10.1371/journal.pone.0297969

**Published:** 2024-02-08

**Authors:** Anneke Zuiderwijk, Berkay Onur Türk, Frances Brazier

**Affiliations:** 1 Faculty of Technology, Policy and Management, Delft University of Technology, Delft, the Netherlands; 2 Education and Student Affairs, Eindhoven University of Technology, Eindhoven, the Netherlands; Macquarie University, AUSTRALIA

## Abstract

To understand how open research data sharing and reuse can be further improved in the field of Epidemiology, this study explores the facilitating role that infrastructural and institutional arrangements play in this research discipline. It addresses two research questions: 1) What influence do infrastructural and institutional arrangements have on open research data sharing and reuse practices in the field of Epidemiology? And 2) how could infrastructural and institutional instruments used in Epidemiology potentially be useful to other research disciplines? First, based on a systematic literature review, a conceptual framework of infrastructural and institutional instruments for open research data facilitation is developed. Second, the conceptual framework is applied in interviews with Epidemiology researchers. The interviews show that two infrastructural and institutional instruments have a very high influence on open research data sharing and reuse practices in the field of Epidemiology, namely (a) access to a powerful search engine that meets open data search needs and (b) support by data stewards and data managers. Third, infrastructural and institutional instruments with a medium, high, or very high influence were discussed in a research workshop involving data stewards and research data officers from different research fields. This workshop suggests that none of the influential instruments identified in the interviews are specific to Epidemiology. Some of our findings thus seem to apply to multiple other disciplines. This study contributes to Science by identifying field-specific facilitators and challenges for open research data in Epidemiology, while at the same time revealing that none of the identified influential infrastructural and institutional instruments were specific to this field. Practically, this implies that open data infrastructure developers, policymakers, and research funding organizations may apply certain infrastructural and institutional arrangements to multiple research disciplines to facilitate and enhance open research data sharing and reuse.

## Introduction

Science is making a paradigm shift towards data-driven research, where data intensity and collaboration have deemed research data sharing a necessity [1,2]. The data that researchers collect, process, and analyze can create value beyond their initial intended purpose when shared “openly” with others on the internet in a freely accessible, usable, modifiable, and shareable format [3,4]. In this study, open research data refers to both qualitative and quantitative data. It concerns data that is actively published on the internet in the public domain for public reuse, and that is freely accessible, usable, modifiable, and sharable by researchers without restriction [5], provided that there is appropriate acknowledgment if needed [6]. Open research data can be raw/primary, derived from primary data for subsequent analysis or interpretation, or derived from existing sources held by others [6]. The benefits of open research data range from increased transparency [7,8] to decreased time and effort spent by researchers on repetitive and unnecessary data collection processes [2,9] to new options for producing scientific knowledge through meta-analyses of different open data sets [10].

However, despite the benefits, researchers often have good reasons for not sharing and reusing open research data. At the *infrastructural level* researchers may not openly share their data due to technical issues [11–13] or data quality issues [8,9,14], and at the *institutional level* researchers may fear a loss of publishing opportunities [15–18], loss of credit [11,15,19,20], or the (perceived) effort required may be too great [9,14,21–23]. At the *infrastructural level*, researchers may not reuse open research data because of a lack of data standardization [24] or because the data may not be Findable [25,26], Accessible [25], Interoperable [25,27], or Reusable [12] (FAIR). At the *institutional level*, data sharing policies may not be sufficiently supported for researchers to be able to acquire open research data use skills [27]. These institutional and infrastructural factors are strongly related and interdependent.

Previous research provides in-depth examinations of both the drivers and inhibitors of data sharing motivations [e.g., 2,14,22,28], often in specific research disciplines, such as Sociology and Political Science [29], Biodiversity [8], Health [12], Natural Sciences [1], Social Sciences [30], and Genetics and Life Sciences [31]. These studies provide insight into the discipline-specific challenges and opportunities for promoting open research data practices within these disciplines. Furthermore, although many factors contribute to researchers’ open research data sharing and reuse motivations, previous research shows that the combination of infrastructural and institutional instruments known as *arrangements* [e.g., 11,32] is promising to facilitate and stimulate open research data sharing and reuse. Institutional instruments concern formal structures (e.g., university policies), informal structures (e.g., norms, culture), and operational mechanisms (e.g., existing data-sharing processes) that research institutions can employ to incentivize open research data sharing and use [derived from 33,34]. Infrastructural arrangements refer to both technical elements (e.g., open data portals, infrastructures, (meta)data standards and formats, and tools for processing, searching, analyzing, and visualizing data, and data quality mechanisms) and governance elements (e.g., mechanisms to enhance privacy, trust, and interaction with other data providers and users) to stimulate open research data sharing and reuse [derived from 35].

As infrastructural and institutional arrangements could also differ across fields, insight is needed on which instruments work well under which conditions in specific research disciplines, and to what extent the instruments in these disciplines differ from one another. One research discipline that has received considerable attention in society over the past few years is Epidemiology. Epidemiology is “the study of the distribution and determinants of health-related states or events in specified populations, and the application of this study to the control of health problems” [36, p. 61]. Previously, researchers have made various types of data relevant for the field of Epidemiology publicly available, such as genomic sequence data [37,38] and data about the determinants associated with the epidemic diseases [39]. There is enormous potential of open research data sharing and reuse in Epidemiology. For example, open research data in this field may provide insights that allow the development of better handling or treatment of adiposity, diabetes, cardiovascular disease, dementia, and other diseases, which as pressing issues that need effective strategies. Also in other situations, openly sharing data derived from studies in Epidemiology can have significant benefits [40–42].

Various steps have already been taken in the field of Epidemiology to improve open research data sharing and reuse. For example, the field of Genetic Epidemiology made considerable progress by applying data sharing for individual patient data meta-analyses [40]. Moreover, many cohort datasets in Epidemiology have been made accessible for reuse by others [e.g., 43,44], leading to better research. Yet, despite some exceptions, open-science practices have been embraced at a slower pace in research in Epidemiology compared to the Social Sciences [41]. To understand how open research data sharing and reuse can be further improved in the field of Epidemiology, this study explores the facilitating role that infrastructural and institutional arrangements play in this research discipline. This study addresses the following research questions: 1) What influence do infrastructural and institutional arrangements have on open research data sharing and reuse practices in the field of Epidemiology? And 2) how could infrastructural and institutional instruments used in Epidemiology potentially be useful to other research disciplines?

Focusing specifically on infrastructural and institutional arrangements in Epidemiology could give open data infrastructure developers, policymakers, and research funding organizations better guidance on how open data sharing and reuse practices can be facilitated and enhanced in this field. Moreover, this study complements scientists’ understanding of field-specific challenges and opportunities for open research data.

## Methods

**Fig 1:**
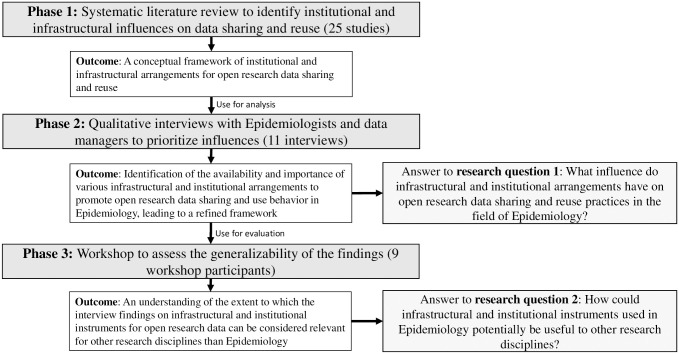
Overview of the research phases and their types of outcomes.

This study is conducted within the context of an interpretivist research paradigm [see 45,46], in which phenomena are examined based on the significance assigned to them by research participants [47]. Interpretive research methods primarily encompass qualitative and participatory approaches with the objective of comprehending situations [46]. Such research is particularly appropriate for scenarios where problems are not fully comprehended, emotionally charged, or within politicized organizational contexts [48]. This study combines three research methods applied in a qualitative manner: a systematic literature review, interviews, and a research workshop (see Fig 1). These methods were conducted by the second author of this article as part of his MSc graduate project at Delft University of Technology in the Netherlands, under supervision of the first and third author. Both the principal investigator and the supervisors received training in terms of qualitative research, including all the involved research methods, and both supervisors had more than ten years of experience in qualitative research methods.

This study was approved by the Human Research Ethics Committee of Delft University of Technology in the Netherlands on 29 March 2022, under application number 2068. Informed written consent was received from all interview participants of this study and verbal consent acquired from all workshop participants. Some participants specified that they did not want their interview transcripts and/or summaries to be shared publicly. Therefore, this data is not available online.

### Phase 1: Systematic review to identify institutional and infrastructural influences on data sharing and reuse

**Fig 2:**
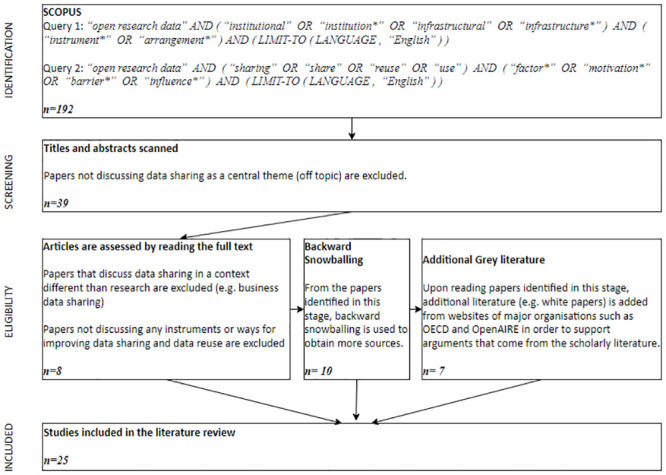
Literature selection and assessment (using the PRISMA flow diagram).

The purpose of the literature review was to identify literature on institutional and infrastructural arrangements for open data research sharing for further analysis and the design of a conceptual framework. The phases of the systematic literature review [see 49], complemented by analyses of grey literature, such as white papers, reports, and guidelines, are depicted in Fig 2.

Of the 192 English papers found in the SCOPUS database (using a broad search approach) in the identification phase, 153 were excluded in the screening phase (in which the title and abstract were read) due to lack of focus on data sharing, 31 during the eligibility phase (in which the papers were read in full) due to lack of relevance to a research context, and only 8 papers remained. Ten additional papers were identified through backward snowballing and seven papers from key organizations involved with the topic of open research data, resulting in a total of 25 studies in which reference is made to infrastructural and institutional instruments that can potentially enhance open data sharing and reuse in general. The final set of references to these included studies is provided in Tables 2 and 3 in the Results Section.

**Fig 3:**
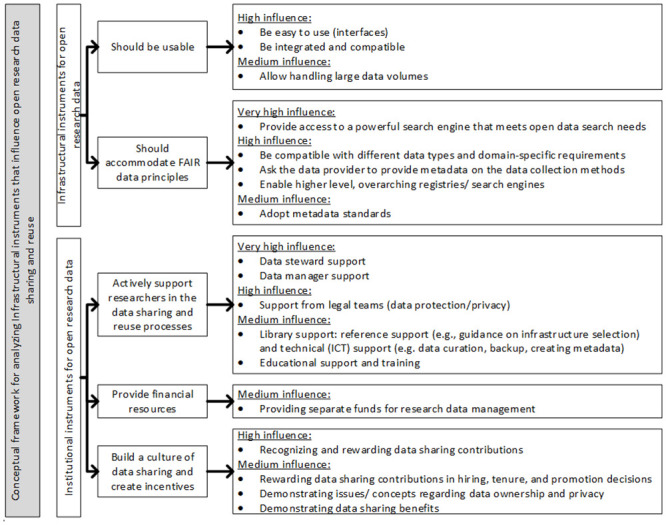
Refined, empirically enhanced framework of infrastructural and institutional instruments influencing open research data sharing and reuse in our study in Epidemiology.

An overview of infrastructural and institutional instruments that may or may not be used in Epidemiology [see 50] was derived from the 25 selected studies. This overview provided the basis with which relevant instruments were grouped together to create a conceptual framework. There appeared to be partial overlap between some of the instruments identified through the literature review. For example, multiple instruments in the literature refer to the ease of use, but in a slightly different formulation. In this case, the instruments were rephrased to better explain in what context the ease of use of data sharing and reuse was addressed, such as the ease of use of the user interfaces or the data analysis. Moreover, some instruments were rephrased because we expected that they would be difficult to comprehend by the study participants and that the rephrased instrument would be more evocative. The conceptual framework (presented later in this article in Fig 3) was then used as a basis for the interview questions in the next phase of our study.

### Phase 2: Qualitative interviews with Epidemiologists and data managers to prioritize influences

Interviews are well suited to view the social world from the perspective of a specific actor and to gain insight into opinions on complex issues [51]. In addition, interviews allow for “enquiring openly about situational meanings or motives for action” [52, p. 203]. Due to these characteristics, interviews were considered appropriate to explore the underlying context-dependent motivations of researchers towards open research data adoption in our study. The focus is on analysis of “why” a certain process (i.e., open research data sharing and reuse) occurs the way it does in a specific research discipline (i.e., Epidemiology) during a specific point in time (namely a period that coincided with the ongoing COVID-19 pandemic) warranting an interview approach. The interviews were focused on open research data sharing and reuse in the Netherlands. The interviewees were from different organizations in the selected country in the defined research discipline with some experience in the use of infrastructural and institutional arrangements to promote open data sharing and reuse. Two policy documents [53,54] and four websites [55–58] provided background information.

To obtain a rich collection of views and perspectives, the main strategy when selecting interviewees was to get as many University Medical Centers involved as possible. The initial criterion was to include at least one researcher from each University Medical Centre in The Netherlands. Multiple rounds of (repeated) email invitations were sent to 105 researchers in distinct Epidemiology Departments found on the University Medical Centers’ websites. In addition, two participants from the personal network of the first author were invited to participate. Due to limited response, not all University Medical Centers are represented in our study (e.g., Erasmus Medical Centre, Maastricht Medical Centre, and Radboud Medical Centre). Of those researchers who declined the interview invitation, all said they did not have the time to participate.

Eleven researchers from five Medical Centers (Leiden University Medical Centre (LUMC), University Medical Centre Groningen (UMCG), Amsterdam University Medical Centre, Utrecht University, and University Medical Centre Utrecht) chose to participate: ten whom work in the field of Epidemiology and one with expertise on legal aspects of open research data practices and research data management. The eleven participants did not know the researcher who conducted the interviews before the interviews took place. The interviewees were informed through email about the researcher’s reasons for doing the study, where the researcher’s strong interest in the research topic was expressed. Table 1 depicts the main characteristics of the interviewees (6 males, 5 females). Altogether, these interviewees covered seven sub-fields in Epidemiology: General Epidemiology, Environmental Epidemiology, Clinical Epidemiology, Pharmaco-Epidemiology, Spatial Epidemiology, Big Data Epidemiology, and Infectious Diseases Epidemiology.

**Table 1:** Background information of the interviewees.

Interviewee no.	Role of interviewee	Age group	Academic position	Experience with open research data sharing and/or reuse
I1	Epidemiology Researcher	25–30	Ph.D. student	Experience with open research data reuse
I2	Epidemiology Researcher (and Policy Advisor)	36–40	Assistant Professor	Experience with open research data sharing and reuse
I3	Epidemiology Researcher	56–60	Full professor	Experience with open research data reuse
I4	Epidemiology Researcher	31–35	Postdoctoral Researcher	Experience with open research data reuse
I5	Epidemiology Researcher	25–30	Postdoctoral Researcher	Experience with open research data sharing and reuse
I6	Epidemiology Researcher	25–30	Ph.D. student	No experience in open research data practices
I7	Epidemiology Researcher	25–30	Postdoctoral Researcher	Experience with open research data reuse
I8	Epidemiology Researcher	41–45	Assistant Professor	Experience with open research data sharing and reuse.
I9	Epidemiology Researcher	41–45	Associate Professor	Experience with open research data sharing and reuse
I10	Epidemiology Researcher	46–50	Associate Professor	Experience with open research data sharing
I11	Research Data Management Consultant	Unknown	N.A.	Expertise in the legal/privacy aspects of open data practices and research data management

The interviews focused on: (1) background information, (2) previous experiences in open research data sharing and reuse, (3) infrastructural instruments that influence motivation and behavior towards open data practices, (4) institutional instruments that influence motivation and behavior towards open data practices, and (5) barriers to open research data sharing and reuse (see Appendices A and B). The conceptual framework guided the way the specific interview questions were phrased. The instruments as included in the conceptual framework were discussed during the interviews, where respondents were asked to reflect on the availability and importance of each instrument. The interview questions can be found in S1 File. A guide for the interview was developed and pilot tested. An initial version of the interview questions was tested in trial interviews with two open data researchers (from another field) and adapted slightly.

The interviews were conducted from March 28 until May 3, 2022. The interview lengths varied from 30 to 90 minutes. The interviews were conducted online, using the Microsoft Teams software. Besides the principal investigator and the interviewee, no one else was present during the interviews. The data was collected based on signed informed consent in which all participants agreed to be recorded. Interview summaries were (1) created by the second author based on automatically generated transcripts (2) reviewed and approved by the participants and (3) inductively and deductively coded in multiple iterative cycles (following the guidelines by Linneberg and Korsgaard [59]). Coding was done manually. The second author conducted the data coding, while this coding process, a sample of coding examples, and cases of doubt were discussed with the first and third author. More detailed information on the coding process can be found in [50]). Moreover, the codebook underlying this study is openly available through the 4TU.ResearchData repository (see http://doi.org/10.4121/20085560, see [60]). Finally, the data saturation was discussed among the three authors and led to the conclusion that no follow up interviews were needed.

Employing the interview transcripts, a counting approach was applied to determine 1) the availability and 2) the importance of the identified infrastructural and institutional instruments for open research data sharing and reuse. As stated by [61], “counting is integral to the analysis process, especially to the recognition of patterns in data and deviations from those patterns, and to making analytic or idiographic generalizations from data” (p. 231). This is also the case in qualitative research as both descriptive and inferential statistical measures can add meaning to qualitative data by transforming them into quantitative data [61]. “Reducing qualitative data to numbers can sharpen the focus on a key finding” (p. 233) by making patterns appear more clearly, and by generating new questions and new lines of analysis [61]. So-called supplementary counting, which is a form of counting that “builds on other findings and adds to them, enabling researchers to develop new insights into their phenomena of interest” [62, p. 16], was used as the intention of supplementary counting is neither to create the central contribution of the research, nor to confirm other findings [62].

To measure the *availability* of each infrastructural and institutional instrument, respondents were asked whether they were able to use or apply the instrument in practice (sections 2 and 3 in Appendix A). For example, for the instrument “*Availability of a search engine that is sufficient for open data search needs*”, the statement was "*The search engine on the open data repository that I use is sufficient for my open data search needs*”. The statements of respondents were classified as “yes” or “no”. Only the responses with a definitive answer were used in further analysis. The respondents who could not give a definitive answer to this question (e.g., because they were not sure) were omitted.

To measure the *importance* of each instrument, the answers given to the interview question “*To what extent does this instrument influence your open research data sharing and reuse behavior*?*”* (See Appendix A) were first classified either as important or not important. An answer is classified as important (*“[the instrument] is an important factor for open data practices”*) if it meets at least one of the following conditions:

- The respondent explicitly mentions that the instrument has an “influence” on open data practices (negative or positive), or that the instrument is “important” or “valuable” or “useful” for open data practices.
- The respondent describes a concrete causal relationship of how the instrument influences open data practices.
- The respondent states that there is a “need” for such an instrument for open data practices or better open data practices.
- The respondent states that they would like to have access to this instrument for their open data practices or that they are “happy” or “satisfied” by already having access to it.
- The respondent states that if this instrument existed, the level of open data practices would be affected.

An answer is classified as not important (*“[the instrument] is* *not* *an important factor for open data practices”*) if it meets at least one of the following conditions:

1. The respondent explicitly mentions that the instrument does not affect open data practices at all, or it affects such practices at low levels.
2. The respondent explains that there is not a relationship between (the existence of) the instrument and open data practices, or that the relationship is highly doubtful or highly questionable.
3. The respondent states that not having access to this instrument is not a (strong) barrier to open data practices.
4. The respondent states that researchers do not need this instrument for their open data practices.
5. The respondent states that researchers are not interested in (using) this instrument regarding open data practices, or that they choose not to use or engage with the instrument even if they have (or could have) access to it.

To determine the extent to which an instrument has an influence on open research data practices, the *difference* between the number of respondents for each classification is determined. Thus:

[*# of respondents who state that having the instrument is an important factor for open data practices—# of respondents who state that having the instrument is not an important factor for open data practices*] = *X*

- If (X) is equal to or smaller than 1, the instrument is considered to have little influence;
- If (X) is 2, medium influence;
- If (X) is between 2 and 5, high influence;
- If (X) is equal to or larger than 5, very high influence.

For examples, please see the codebook [60]. Please note that, although this research is qualitative, this operationalization is quantitative, and that this analysis is based on a small sample.

After applying the above-mentioned counting approach, the identified infrastructural and institutional instruments were ranked in terms of availability and importance for open data sharing and reuse. The objective of this ranking was to select instruments to be discussed in further detail in the next phase of our research, namely during a research workshop. Thus, the ranking was used as a manner to prioritize the most available and the most important infrastructural and institutional instruments used in Epidemiology, according to our interviewees.

### Phase 3: Workshop to assess generalizability of findings

As workshops enable participants to interact and collaborate while learning about a topic, with collaboratively shared learning experiences, they can also provide valuable information or artefacts that would not be possible to obtain from other research methods [63–65]. In this study, our main rationale for conducting a research workshop was to acquire an understanding of the extent to which the interview findings on infrastructural and institutional instruments for open research data can be considered relevant for other research disciplines than Epidemiology.

A guide created specifically for this study, prescribed the elements of the workshop and the questions to be discussed, including the workshop’s purpose, how the workshop was to be conducted, the questions themselves, the important instruments to be evaluated during the workshop, and a final short survey. A one-hour interactive workshop was conducted with nine participants from one Dutch university who either work as a data steward or a research data officer and have different backgrounds (Mechanical Engineering, Software Engineering, Astrophysics, Social Sciences, Computational Physics, Geology, Information Management, Microbiology, and Molecular Biology and Genetics). The workshop was conducted online using MS Teams. The participants did not know the workshop organizer before the workshop took place. They were approached through email and received information about the principal investigator’s main motivations for the study. The participants first followed a twenty-minute presentation on the research objective, research approach, and our interview findings. To explore the question “*Which infrastructural instruments (found in this study for Epidemiology) could potentially be useful for open research data sharing and reuse in other fields and why*?*”*, participants were given five minutes to individually describe their comments on the instruments using an online collaborative whiteboard platform (Miro, 2002) after which a group discussion was held where the participants shared their input with the rest of the group. This procedure was repeated for the same question for institutional instruments. Then, in a short survey, workshop participants were asked to rank the infrastructural and institutional instruments, just as had been requested from the interviewees. With consent of the participants, the workshop was recorded. Besides the principal investigator and the participants, no one else was present during the workshop. After the workshop, the findings were discussed among the authors, revealing data saturation.

## Results

### Infrastructural and institutional instruments identified

This section provides the results of our systematic literature review on infrastructural and institutional instruments for open research data adoption (research phase 1). Twenty generic infrastructural and 26 generic institutional instruments for open data sharing and reuse were identified through our literature review (Tables 2 and 3).

**Table 2:** Overview of infrastructural instruments derived from the literature review.

Instrument categories	Instruments	Sources mentioning these instruments
**Instruments enhancing the usability of open research data infrastructures**	Accommodate large-scale data, should be easy to use, should enable an increase in data storage, and should be reliable	Campbell [25], Harper and Kim [18], Kim and Adler [22], Zuiderwijk, Shinde [66]
	Actively supporting researchers and incorporating licensing and copyright processes	Patel [7], Piwowar, Day [67], Schmidt, Gemeinholzer [20]
	Infrastructures should be integrated and compatible	Behnke, Staiger [68], Koski, Gheller [69], van Gend and Zuiderwijk [35]
	Data repositories should be easy to use, and should have user-friendly graphic interfaces	Behnke, Staiger [68]
	Data repositories should enable the researcher to do data analysis (as an integrated feature)	da Costa and Leite [23]
	Infrastructures should offer assistance in the choice of repository	Downs [70]
	Availability of Research Data Management tools (e.g., DMPTool and DMPonline).	Michener [71]
**Instruments supporting the facilitation of FAIR research data principles**	The data repository accommodates and incentivizes the usage of metadata standards: it can store metadata and enable researchers to browse metadata.	Shelly and Jackson [72], van Gend and Zuiderwijk [35], Zuiderwijk, Shinde [66]
	Availability of software/tools that are used for metadata creation and management.	Michener [71]
	The open data repository stores (meta)data on data collection methods and enables browsing.	Michener [71]
	The data repository inflicts and accommodates proper data citation (standards) so that data can be easily found and attributed.	Crosas [73], Patel [7]
	Compatibility with different data types and different domain-specific requirements.	van Gend and Zuiderwijk [35], Zuiderwijk, Shinde [66]
	Infrastructures are linked to higher-level search engines/ registry of repositories that enable researchers to search data across different data repositories easily.	Behnke, Staiger [68], van Gend and Zuiderwijk [35]
	Providing various query interfaces to accommodate different data search behaviors for the searching functions in infrastructures.	Behnke, Staiger [68]
	Data usage statistics should be made available on the infrastructures.	Behnke, Staiger [68]
**Instruments concerning security and trust aspects of openly sharing and reuse research data**	Application of certification instruments	Downs [70]
	Availability of data anonymization tools	Shelly and Jackson [72]
	Design against accidental data loss	Patel [7]
	Infrastructure should be secure against breach	Patel [7]
	A variety of access restrictions should be possible on the infrastructure	Behnke, Staiger [68]

**Table 3:** Overview of institutional instruments derived from the literature review.

Instrument Category	Instrument	Sources mentioning these instruments
**Instruments that manage and govern open research data sharing and reuse processes**	Establishing concrete data management plans	Michener [71], Shelly and Jackson [72], Tenopir, Birch [74]
	Establishing institutional data sharing policy and guidelines for data sharing	Michener [71], Shelly and Jackson [72], Patel [7]
	Establishing policies for research data security	Patel [7]
	Ensuring that researchers think about costs related to access, management, and preservation of data before the research starts.	Organization for Economic Co-operation and Development [OECD] [75]
	Establishing a data deletion policy.	Behnke, Staiger [68]
	Giving researchers a clear legal basis about rights of use, so that they understand what they are allowed to do with the data; explaining legal requirements and options of compliance to such requirements; asking the researcher to clarify terms of use at the beginning of the research cycle (e.g., concerning licensing, privacy confidentiality).	Fecher, Friesike [14], Patel [7], van Gend and Zuiderwijk [35], Delft University of Technology [76]
	Supporting the alignment of organizational data sharing and management policies between organizations and countries.	Clarke and Davidson [77]
	Giving a clear guideline on how to obtain consent for data sharing.	Fecher, Friesike [14]
	Giving a clear guideline on how to anonymize data.	Fecher, Friesike [14]
**Instruments that actively support researchers in sharing and reusing open research data**	Establishing support from libraries, clarify the role of libraries.	Neylon [78], Shelly and Jackson [72], Tenopir, Birch [74], Zuiderwijk, Shinde [66]
	Providing guidance on the selection of data repository as early as possible in the research cycle.	Downs [70], van Gend and Zuiderwijk [35]
	Establishing support from the legal teams of the organization.	Scholtens, Anbeek [79]
	Placing practical information (e.g., guides on locating data or funding agency requirements) on webpages and web guides; carrying information from traditional information platforms (e.g., documents) to web pages.	Neylon [78], Shelly and Jackson [72], Tenopir, Birch [74], Zuiderwijk, Shinde [66]
	Educational support on data management: providing training to researchers	Neylon [78], Shelly and Jackson [72], Tenopir, Birch [74], Zuiderwijk, Shinde [66]
	Availability of data stewards whose roles are concretely established in the organization.	Scholtens, Anbeek [79]
	Possibility of working with data managers to shift responsibility from researcher to an experienced professional.	Utrecht University [80], van Gend and Zuiderwijk [35]
**Instruments that relate to financial resources for openly sharing and reusing research data**	Providing financial support to researchers and make the availability of funding clear.	da Costa and Leite [23], Piwowar, Becich [81], Zuiderwijk, Shinde [66]
**Instruments that build a culture of research data sharing and reuse and create incentives**	Revising policies and guidelines in an institution to reflect data sharing goals	Patel [7], Piwowar, Becich [81]
	Recognizing and rewarding data sharing contributions (e.g., via track metrics)	Piwowar, Becich [81], van Gend and Zuiderwijk [35]
	Data sharing contributions should be considered during hiring, tenure, and promotion decisions.	Piwowar, Becich [81]
	Implementing data citation policies	Mooney and Newton [15]
	Demonstration of benefits of and needs for data sharing	Piwowar, Becich [81]
	Demonstration of how the issues around data ownership and privacy can be tackled.	Piwowar, Becich [81]
	Creating incentivizes from publishers or from organizations (e.g., requests for sharing data)	Michener [71], Piwowar, Becich [81], Zuiderwijk, Shinde [66]
	Creating incentivizes from funders (e.g., requests for sharing data or by evaluating a proposal’s data sharing plan under its scientific contribution)	Michener [71], Patel [7], Piwowar, Becich [81]
	Actively publishing experiences in data sharing to incentivize researchers	Piwowar, Becich [81], van Gend and Zuiderwijk [35]

### Interview participants’ identification and prioritization of instruments

From the identified generic infrastructural and institutional instruments for open data sharing and reuse, a conceptual framework was derived that included fifteen infrastructural instruments and fourteen institutional instruments (see Tables 5 and 6). All instruments can be classified as being primarily related to usability, FAIR data principles, or to security and trustworthiness. The framework provided the basis for the interviews conducted with professionals working in Epidemiology to prioritise these instruments by their influence on data sharing and reuse in Epidemiology. During the interviews, three new infrastructural instruments and two new institutional instruments were identified (see Tables 5 and 6). Six infrastructural instruments and four institutional instruments were found to have a high or very high influence on Epidemiological data sharing and reuse. The interview results regarding these highly influential instruments are outlined below (research phase 2), answering our first research question: *What influence do infrastructural and institutional arrangements have on open research data sharing and reuse practices in the field of Epidemiology*? In the following analysis, note that quotes from individual interviewees are indicated by an interviewee number between square brackets (for example, [I1] refers to the first interviewee in Table 1).

#### Infrastructural instruments of high or very high influence

Table 4 depicts the availability and importance of the infrastructural instruments included in the conceptual framework for openly sharing and reusing research data in the field of Epidemiology. The last column depicts the level of influence of each infrastructural instrument on data sharing and reuse in our study. The instruments with a “very high” and “high” influence, are discussed referring to [50] for an extensive discussion of the other instruments.

**Table 4:** The influence of the examined infrastructural instruments in Epidemiology (see Methods section for the operationalization).

Infrastructural instrument category	Identified infrastructural instruments	Availability of the instrument		Importance of the instrument		The influence on data sharing and reuse in our study
		# of respondents who say they use the instrument	# of respondents who say they do not use the instrument	# of respondents who find the instrument important for open data practices	# of respondents who find the instrument unimportant for open data practices	
**Instruments enhancing the usability of infrastructures**	Easy to use, convenient interfaces	5	2	4	0	High
	Capability to handle a large volume of data	5	2	3	1	Medium
	Compatible and/or integrated infrastructures	3	5	5	1	High
	The data repository allows for data analysis (integration)	2	3	1	2	Low
	Availability of data management tools	4	1	1	0	Low
**Instruments supporting the facilitation of FAIR data principles**	Availability of a search engine that is sufficient for open data search needs	2	5	6	0	Very high
	Availability of higher-level search engines/registry of repositories that enable researchers to search data across different repositories	0	5	4	0	High
	Availability of data usage statistics on the platform	1	3	0	3	Low
	The infrastructure offers metadata	6	1	2	0	Medium
	The infrastructure offers metadata on data collection methods	-	-	4	0	High
	Availability of tools that are used for metadata creation and management.	0	2	1	0	Low
	Offering assistance for data citation	2	0	-	-	Unclear
	The infrastructure is compatible with domain-specific privacy requirements	0	2	3	0	High
**Instruments concerning security and trust aspects**	Availability of data anonymization tools	1	4	0	3	Low
	Offering ways/methods to assess how trustworthy an open data repository or an open data set is.	0	3	-	-	Unclear
**New instruments detected through the interviews**	Fast download process	-	-	2	0	Unclear (mentioned by two respondents)
	Standardize way of working among different repositories	-	-	1	0	Unclear (mentioned by one respondent)
	Enhance the usage of unique identifiers, such as the ORCID identifiers	-	-	1	0	Unclear (mentioned by one respondent)

Only one infrastructural instrument was shown to have a very high influence on open data sharing and reuse practices, namely, the “*availability of a search engine that is sufficient for open data search needs*”. During the interviews the majority of the interviewees stated their dissatisfaction with the current search engines on the repositories, citing major problems regarding the findability of research data. [I2], for example, states that in their experience finding data has been a major problem and thinks that not being able to find the data is definitely a barrier for open data practices. If researchers know what they are looking for (i.e., if they have a Digital Object Identified [DOI] at hand) then reaching the data is straightforward, but if they have to “search” for data, then there are difficulties because there is no particular way to search for data on a specific topic [I2].

During the interview, [I2] demonstrated an exemplary search on the open data repository Zenodo, where they typed an Epidemiology related keyword, and showed that many of the results are results that are completely unrelated to the search (most results are what [I2] calls “junk” that inhibits motivation for open data reuse). [I7] also cites the same problem with the Zenodo platform: *“I can never find the stuff I need on it*.*”* [I7]. [I9] mentions the problem of insufficient search engines as the most troublesome aspect of open data infrastructures such as GitHub, citing the same reasons: “*That’s maybe the most difficult thing is that […] we’re very accustomed to PubMed or Google right to find articles or find information*. *But for finding data*, *a proper search engine for finding data that relates to your question*, *is there a search engine like that*? *Do you know of a search engine that does that*? *[…] So that makes it really*, *actually unfindable*.*”* [I9].

Five instruments were shown to have a high influence on openly sharing and reusing research data in our study:

- *Easy to use*, *convenient interfaces*. Several interviewees noted this instrument as an important factor for open data sharing practices. [I9] and [I10] had issues in the past with dealing with the interfaces of the open data repositories, both reporting the issue of not being able to find the dataset they uploaded to the data repository. To quote [I9]: “*A journal wanted me to upload all my data […] It was complicated*. *[…] It kind of inhibited me from doing it [sharing the data openly] almost*. *[…] I didn’t understand [how to upload the data] […] Then it said I had successfully uploaded*. *[…] I couldn’t find my own data*.*”* [I9]. As far as open data reuse is concerned, many participants who reused datasets from repositories state that the (graphic) interfaces are user-friendly and convenient to use. [I2] states that data reuse is just a matter of getting a CSV file from the repository or through an API, so there is no issue for ease of use.
- “C*ompatible and/or integrated infrastructures*”. The compatibility and integration between different data structures are perceived as an important factor for open data practices by many researchers [I1, I5, I7, I8, I9, I10]. However, the interviewees indicate that currently, the level of compatibility and especially the integration between infrastructures used for open data practices in Epidemiology are not at satisfactory levels. [I8] and [I4] state that (full) integration is currently not a reality, but, for [I8], compatibility is an essential element for open data practices. [I2] gives an example of some infrastructural integrations that they perceive to be helpful, such as how Open Science Framework (OSF) is well integrated with Github, with storage applications like Dropbox, and with discovery applications like Google Scholar and ORCID. [I7] also mentions the integration between Zenodo and Github. Several interviewees (I4, I7, I10) express their dissatisfaction regarding the compatibility between different infrastructures, especially compatibility issues due to data types. [I10] indicates that Epidemiology researchers struggle because “…*we work with R most of the time*, *and some data sets are easy to upload into your environment*, *like a*.*CSV file […]*. *But nowadays more and more data that is collected through an internet interface like apps -we use apps to monitor persons*, *etcetera-*, *they come in XML files*, *and that is more complicated*. *[…]”*. [I10] suggests that there is a relationship between certain compatibility issues and the need for training that researchers need to receive to be able to engage in open data practices: *“A lot of this type of work -where people would start working with [reusing] data sets from somewhere-*, *is done by PhD students*. *Now*, *you almost need to have a background in computer science to be able to deal with all the different types of data sets and to get that in your statistical analysis software environment*. *[…] So*, *it requires a new set of skills […] which is not typically what you learn in your masters [in] Epidemiology”* [I10].
- “*The availability of higher-level search engines/registry of repositories that enable researchers to search data across different repositories*”. Several interviewees state that the individual repositories that they use are not linked to any aggregator infrastructures where it would be possible to search for data across resources [I1, I2, I5, I7]. [I2] states, *“There’s no way to search for everything [meaning across repositories]”* [I2]. Furthermore, they express the need for such an aggregating search tool and believe that this is an important factor for open data practices. [I9] states that researchers should not have to go to Google (databases) when they want to find a dataset belonging to a certain demographic in a specific region. Instead, there should be a search engine available for this (which [I10] calls, for example, “a PubMed for research data”), to where all the data repositories are linked, and this infrastructure should print the datasets linked to your keywords or your extensive search queries [I5, I9, I10]. [I11] states, *“I think that there’s not a good open research data search engine yet*. *You have Google databases*, *you have a few [engines] here and there*, *but*, *for example*, *one of the famous repository search engines where you look for repositories*, *it is abysmal*. *I do not recommend it*. *Half of the links are broken*. *I think that there is definitely a niche or a spot there to be filled by a proper research data search engine*.*”* [I11]. The availability of an overarching search engine/registry is found to have a high influence on open data practices in our study.
- *The infrastructure offers metadata on data collection methods*. Although many interviewees state that it is currently possible to find metadata on the open data infrastructures, the current problem with metadata on these platforms seems to be about their content rather than their mere existence. [I1] states that being able to see how each variable was measured and what was asked exactly when the data were gathered is very important. When discussing their data sharing practices, [I2] states that rather than making their dataset fully open, they would prefer to invite data requesters to visit them for two or three days, because the database may be complicated; by doing this, it is possible to inform data requesters on the variables. [I2] adds that they do this because of interpretation: they do not want their study to be wrongly analyzed by others because of an incomplete understanding of the data. [I5] also brings attention to how the lack of such metadata may be an important barrier to open data practices: *“[If] you’re not too sure how data is collected… These kind of things [demotivate]*. *What that variable means*? *If it’s measured with this instrument or with another instrument…”* [I5]. [I9] mentions the same issue about metadata on data collection: “*If somebody says*, *‘this weight of a person’*, *was it measured*, *was it self-reported*? *If it’s not mentioned*, *then how am I supposed to know how it was measured*? *[…] That’s the problem with data dictionaries*, *they don’t [do that]*. *[…] So*, *then you have to go back to the researchers [who prepared the data]*. *[…] It is difficult”* [I9]. The instrument is found to have a high influence on open data practices.
- *The infrastructure is compatible with domain-specific privacy requirements*. Many researchers noted privacy regulations as one of the strongest barriers to open data practices. Relating to this, the open data infrastructure’s compatibility with (domain-specific) privacy requirements (as an instrument) is also named to be of high influence on open data practices. [I7] states that the existence of privacy rules leads to the necessity of these technical systems to gain new features that enable the accommodation of sensitive data, without violating privacy legislation. [I9] brings attention to the fact that it is currently really hard to link datasets to overarching registries because the data repositories do not give any easy space to deal with privacy regulations, which suggests that researchers are somewhat expecting support from infrastructures on how to overcome issues stemming from privacy regulations. [I7] states, *“It is quite hard actually for us in this field to share data because it’s often patient level*. *So*, *I do feel like especially for medical data sets -patient level data sets- that if we would want that to be more open*, *you would need some kind of [an] infrastructure*. *I don’t think any of the infrastructures that are out there right now cater to this kind of data and therefore*, *when you talk about sharing your data in a repository or online or anything*, *everybody just tells you can’t really do it*. *[…] People are just saying no because of the privacy rules*. *[…] And I think sometimes that’s a bit of a shame*, *because there might be actually ways to work around it*, *but there isn’t really anything [any infrastructure] facilitating that at the moment”* [I7]. Interestingly, some interviewees provide examples of infrastructures and concepts that can address these concerns. One such example is the OpenSafely initiative. OpenSafely infrastructure (although not a fully open data platform by definition) allows researchers to access sensitive data without breaching privacy [82]. Researchers use dummy data for developing their analytic code on their local computer and using the code, they can perform the analysis on the data, without ever accessing the data (that always stays in the secure environment) [82]. [I11] also talks about a similar concept: *“If you [a researcher that wants to work with a certain dataset] have a particular analysis that you want to do on the variables [that you are interested in]*, *then you can send the analysis to the people that currently control the data*. *They can do the analysis and give you back an aggregated result*, *which is then anonymized*. *[…] You can automate this to some degree*.*”* [I11]. Infrastructures like OpenSafely safeguard against important privacy issues, because patient-level data are never seen. This functionality can be very important for data practices in Epidemiology.

These (very) high influence infrastructural instruments are all in the categories of “instruments enhancing the usability of infrastructures” and “instruments supporting the facilitation of the FAIR data principles”, that appear to have an important role in facilitating open research sharing and reuse in Epidemiology. The influence of other instruments was medium (2), low (5), or inconclusive (5).

#### Institutional instruments of high or very high influence

Table 5 depicts the availability and importance of the institutional instruments included in the conceptual framework for openly sharing and reusing research data in the field of Epidemiology. The last column depicts the level of influence of each infrastructural instrument on data sharing and reuse in our study. The instruments with a “very high” and “high” influence, are discussed referring to [50] for an extensive discussion of the other instruments.

**Table 5:** The influence of the examined institutional instruments in Epidemiology (see Methods section for the operationalization).

Institutional instrument category	Identified institutional instruments	Availability of the instrument		Importance of the instrument		The influence on data sharing and reuse in our study
		# of respondents who say they use the instrument	# of respondents who say they do not use the instrument	# of respondents who find the instrument important for open data practices	# of respondents who find the instrument unimportant for open data practices	
**Instruments that manage and govern data sharing and use process**	Offering institutional data sharing policy and guidelines for openly sharing and reusing research data	8	3	1	4	Low
	Offering institutional data management policies	7	1	1	1	Low
	Asking for data management plans	9	0	2	3	Low
**Instruments that actively support researchers in sharing and using research data**	Support from data stewards	3	4	5	0	Very high
	Working with research data managers	5	5	5	0	Very high
	Enhance the library’s role	3	4	3	1	Medium
	Training and educational support	5	1	2	0	Medium
	Providing support for legal aspects (privacy) of open data practices	5	1	4	0	High
**Instruments that relate to financial resources**	Providing separate funds for research data management	0	5	2	0	Medium
**Instruments that build a culture of data sharing and create incentives**	Recognizing and rewarding open research data sharing contributions	0	6	4	0	High
	Considering data sharing contributions during hiring, tenure, or promotion decisions	0	6	2	0	Medium
	Requests for open research data sharing from organizations, funders, journals	10	0	1	1	Low
	Demonstration of benefits of data sharing and the need for data sharing	0	2	2	0	Medium
	Clarifying the concept of data ownership	5	1	2	0	Medium
**New instruments detected through the interviews**	Building an open science community within the Epidemiology field	-	-	2	0	Unclear (mentioned by two respondents)
	Increase communication among the scientific community so that two people with similar research interests are aware of each other	-	-	1	0	Unclear (mentioned by one respondent)

Two institutional instruments were shown to have a very high influence on open data sharing and reuse practices. The first highly influential institutional instrument concerns the “s*upport from data stewards”*. [I1] states that data stewards are there to answer questions in their organization, and that receiving support from data stewards is important because oftentimes there are problems with dealing with data that are hard to understand. [I10] brings attention to how the role functions as a point of referral when you need help: *“It’s [data steward] more approachable*, *I would say*, *[…] And then if they don’t know [how to help]*, *they can send you to someone else*. *As an entry point*, *it’s useful*.*”* [I10]. However, not every organization has a dedicated data steward role ([I2, I3]). Some interviewees are not sure whether they have data stewards to who they can refer for data-related questions [I4, I5], Other interviewees were not sure about the exact role of a data steward. However, despite this, [I3] states that they have colleagues who have a lot of knowledge on data-related subjects and [I3] can easily go and ask these colleagues questions. [I2] states that the policy in their organization is that instead of having a fixed data steward, everyone (individual researchers) is responsible for upholding that stewardship. The majority of the interviewees express the need to be able to refer to a person of contact when they need help.

[I11] confirms that there is currently a *“lack of knowledge about the fact that there is support available*” [I11]. If more researchers know that they can indeed get support for problems they are facing, this could stimulate open data practices [I11]. Researchers who have more experience with open data practices have stronger opinions about the importance of the role of a data steward for open data practices: [I9] explicitly calls the role of data stewardship essential for open data practices. [I8] states that they currently do not receive enough support from the data stewards in their organization, and that the support from the data stewards should be enhanced to reach better open data practices. [I7] states: “*I don’t think it’s [the support from data stewards] sufficient*, *[…] because they’re so busy*, *they don’t have time to*, *personally*, *properly look at the data that you have […]*. *If you have a very straightforward data set*, *then that’s okay*, *because […] they’ve done probably hundreds of cases*, *but the moment your data set is a bit more complicated*, *or there is anything that’s not standard*, *I think they don’t have enough time to properly support you in case you would want to do something*, *for example*, *anonymization*.*”* [I7]. [I7] then gives an example of an incident where they wanted to anonymize a certain dataset to make it open, but they did not get enough support to deal with this procedure. There also seems to be some confusion about the role of a data steward. Several researchers expect data stewards to work on specific datasets or individual projects in detail (e.g., for data anonymization), while traditionally, data stewards do not take these roles in universities (formally).

The second highly influential institutional instrument concerns “working with data managers”. Data managers are reported to be the primary agents that “look after” the datasets and keep them “up to date”, which suggests that their role is vital in ensuring the data can be reusable for open data practices [I7]. However, many interviewees state that they cannot work with data managers [I1, I2, I4, I5, I7], because there are no financial resources available to this purpose. [I7] states they had tried including a data manager in their study before but could not do this due to financial issues. In a few departments, the departments themselves hire a data manager to work for the entire department by allocating their time to different projects [I2, I9].

[I4] states that it is only possible to hire data managers if there is enough research funding. [I2] notes that: *“They [our data managers] only work on making sure that the data that we collect gets in the datasets*. *That’s their level of activities because we can’t pay them*. *[…] But we don’t have money to do the next step on ‘opening up’ the datasets*, *even if we wanted to*.*”* [I2]. In the interviews, apart from having insufficient financial resources, no other reason is reported. [I2] also adds that the (open) research data management activities are not hard to learn, and the topics can be understood in a couple of courses.

Researchers in more senior roles and who participate in larger studies seem to have more access to data managers. [I3], [I8] and [I9] express their satisfaction in being able to work with data managers for building the databases and other specialized data work (e.g., imports/exports of data). [I4] states that there is a culture in their organization that: *“Well*, *if you have research money [you can have data managers]*. *But I’m not sure if I could get money from my department to do that*. *[…] I do see that there is a […] culture*: *[…] if you have a big project*, *you need a data manager*. *[…] But you need research money to do that”* [I4]. [I5] also confirms that hiring a data manager may be a possibility for larger projects, and adds that if more financial funds existed, this would positively affect open research data reuse, because data managers shape the data into standardized formats (which make them reusable). [I4] also states that a barrier to open research data practices is not having money to hire people for research data management activities. [I10] states that nowadays, if they write a grant application, they reserve a budget for research data management. [I7] states that the reason why grants often do not include money for data management activities is that data management is seen as a burden: “*I think data management is something that people*, *still*, *view sometimes as a burden and something that you have to tick the box and then you can go on your merry way*. *And that’s also why often in grants […] there’s not money requested specifically for this kind of people to have them [data managers] on your project*.*”* [I7]

Two other instruments were shown to have a high influence on openly sharing and reusing research data in our study, including “providing support for legal aspects (privacy) of open data practices” and “recognizing and rewarding open research data sharing contributions”. First, the obligation to abide by privacy regulations (i.e., complying with the GDPR) is cited as a strong barrier to open research data sharing in the interviews. Several researchers state that their organization provides some level of support for understanding and fulfilling these legal obligations regarding openly sharing or reusing research data [I5, I7, I8, I9, I10]. For example, [I5] states that there is one person in their team actively checking compliance with privacy regulations. The responsibility of checking whether the GDPR is in compliance falls under the data privacy or data security officers in the organizations [I2, I9]; and researchers report having engaged with privacy officers when they needed support on legal aspects of data sharing.

The instrument of providing support for legal aspects of open data practices has a high influence on open data practices in our study for two reasons. In our analysis of the influence of this instrument, our first finding is that researchers think inadequate assistance is given from their organizations. Due to resource (e.g., time) restrictions, in practice, it is questionable how much useful support the privacy officers currently give to the researchers. [I2] states, *“So in reality*, *it’s difficult to get useful information from them [privacy officers] because there were only two or three of them*. *And they’re overloaded with work”* [I2]. The second finding is that several researchers explicitly stated that their engagements with legal teams or privacy officers in their organization often result in negative outcomes (i.e., the data not being (openly) shared), and that they feel as if the legal teams are not really supportive towards (open) data sharing [I7, I8]. [I7] states that researchers in their organization may have a will to share their research data, but legal teams seem to always focus on the “negative” or focus on giving a “no”, since they always see issues with privacy: *“It’s just that they are very strict on the legal issues*. *They are the ones that are saying*, *‘you should have had informed consent from everyone before you can do anything with your data’ […] They’re trying to make sure that there is no liability at all*, *which I understand*, *but that also makes it very difficult as a researcher […]*.*”* [I7]. [I8] states that: *“They want me to adhere to legal guidelines*, *but at the same time they make it unbelievably difficult for me to do that”* [I8].

The second institutional instrument with a high influence on open data sharing and reuse is “recognizing and rewarding open research data sharing contributions”. The majority of the interviewees state that data sharing contributions are not recognized or rewarded in the field of Epidemiology. [I5] states, “*I think there is no recognition at all*. *[…] There’s no recognition for anything but writing papers basically in my field*.*”* [I5]. [I8] states that they wish there was more recognition for data sharing efforts as these activities make up a large part of their work. Several interviewees state that more rewarding and recognition could lead to higher open data practices in the field. [I10] states, “*I would say that’s [recognition and rewarding for (open) data contributions] what everyone wants it to be like*. *What is now rewarded is publications in high-impact journals*, *and […] the number of citations*, *etc*. *And I think the field really wants to move towards [having] the number of data sets that you have provided for open access and the number of times those data have been reused as a sort of a metric*. *But it’s not really there yet*. *It’s moving slowly”* [I10]. In our study, interviewees state that they are not aware of any track metrics that incorporate data sharing contributions in the field of Epidemiology [I1, I3, I5, I6, I7, I8, I9].

The influence of other instruments than the four highlighted above was medium (6), low (4), or inconclusive (2). These (very) highly influential institutional instruments are all in the categories of “instruments that actively support researchers in sharing and using research data” and “instruments that build a culture of data sharing and create incentives”, which appear to have an important role in facilitating open research sharing and reuse in Epidemiology.

### Refined framework of infrastructural and institutional instruments

The conceptual framework based on the literature has been refined and enhanced based on the interview findings. This led to the framework as depicted in Fig 3. The instruments of low or no influence have been deleted from this framework.

### Generalizability of instruments

The prioritized infrastructural and institutional instruments identified in the previous phase of the research were discussed in further detail during the research workshop. This section discusses the research workshop findings (research phase 3) and addresses the second research question: *How could infrastructural and institutional instruments used in Epidemiology potentially be useful to other research disciplines*? For nine specific findings from our interviews, namely the instruments that have a medium, high, or very high influence, Table 6 presents the workshop participants’ suggestions concerning the relevance of our study findings in other research fields and contexts, as well as their suggestions in the context of the interview findings, that were mentioned during the brainstorm sessions during the workshop. The table shows that none of our interview findings is specific to the Epidemiology discipline. For instance, various other disciplines work with large amounts of data (e.g., Geophysics), require support for legal aspects (e.g., Sociology), and benefit from educational support and training on technical aspects of data sharing (e.g., Electrical Engineering, Mathematics, and Computer Science). The workshop participants stated that some of our interview findings apply to all other research disciplines (e.g., the lack of good search engines, the lack of legal support, and the importance of demonstrating the benefits of openly sharing data) or to many other disciplines (e.g., the lack of sufficient budget for working with data managers, the difficulty of using open data repositories, the usefulness of providing open data repositories providing standardized metadata, and the expectations that data stewards work on specific datasets or individual projects in detail).

**Table 6:** Overview of the workshop findings concerning how infrastructural and institutional instruments used in Epidemiology could also be relevant in other research disciplines.

Interview findings (In the context of Epidemiology)	Relevance of the finding in other research disciplines or contexts	Suggestions in the context of the study’s findings
The infrastructure’s capability to handle a large volume of data (in a timely manner) is important for open data practices. However, Epidemiology researchers struggle with accessing this capability.	In the field of *Geophysics*, some instruments generate a large amount of data, which in practice causes problems with findings repositories that accommodate these data cheaply and effectively.	
The lack of good search engines inhibits the findability of research data in Epidemiology.	This is a problem for *the overall scientific community*. Currently, all fields suffer from the lack of satisfactory engines.	Alternative methods should be developed to compensate for the suboptimal search engines. *However*, building sufficient search engines may not be possible.
Epidemiology researchers get demotivated from open data practices because the available data repositories are not always easy to use during the process of downloading or uploading data.	This could be relevant for *all the fields that do not necessarily collect primary data but also frequently get data from other sources*, because the high number of data resources causes problems with hardships of usage.	Data infrastructures should converge: “Standardize way of working among different repositories”
The open data repository’s offering of standardized metadata is an important motivator for open data practices in Epidemiology.	This is relevant for *all research fields*, as this instrument makes the data search easier, and it makes the data interoperable with other datasets in all research fields.	Data archives should allow for disciplinary metadata to be added to the citation metadata and should provide guidance on how to archive extra metadata items.
Providing timely, structured support for legal aspects (e.g., privacy) of open data practices is important for open data practices in Epidemiology	Providing legal help is relevant for *all fields that deal with personal data*. In these fields, legal procedures in practice take a lot of time, and a lack of timely support is indeed a demotivator.	-Legal experts should help not only focus on only privacy but also on other types of legal issues such as licensing. -Focus on tackling misunderstandings or lack of communication (e.g., legal experts speak a different ’language’ compared to that of researchers, or there is a lack of templates).
Offering educational support and training on technical aspects of data sharing is an important factor for open data practices in Epidemiology	In practice trainings on (open) data sharing prove to be useful in other fields such as Electrical Engineering, Mathematics, and Computer Science. In practice, trainings prove useful when the background of the researcher does not match the requirements of the field in the context of data sharing.	- Focus on clearly identifying the skills that researchers should have and the skills that other RDM professionals should have -Establish a “common base” of education with standardized curricula However, the effectiveness of educational support should not be overestimated: it is hard to provide organizational-level educational support because every researcher or every research project has different situations, and the need to be fulfilled by trainings is very different.
Demonstrating the benefits of data sharing is important for open data practices in Epidemiology	Demonstrating the benefits of data sharing is relevant to *all research disciplines*, but hard to achieve especially in the case of qualitative research and qualitative data (in *all research fields that handle qualitative data*).	
Epidemiology researchers expect data stewards to work on specific datasets or individual projects in detail, although traditionally these roles are not taken by data stewards.	In practice (*in all fields*), the role of a data steward is not geared towards working on specific datasets or individual projects in detail, and these responsibilities are to be taken by data managers in research organizations.	Build a clear distinction between roles (e.g., between data managers and data stewards), so that researchers know who to refer to when they need help.
There is not enough budget for enabling Epidemiology researchers to work with data managers in the context of open data sharing.	The (lack of) sufficient budget issue is an issue in *many other disciplines*: only in the fields where data sharing is really common (e.g., *genomics* research), there is usually a budget allocated for data sharing purposes.	Create a working system that allows researchers to work with a data manager as much as they need, even if only small amounts of time are needed.

Finally, our research participants ranked the institutional and infrastructural instruments, and the ranking showed their preference for institutional instruments over infrastructural ones. However, note that this workshop’s participants, who are mostly data stewards, all work on the institutional side of the problem evaluated in this study. Therefore, the fact that they ranked the institutional instruments over infrastructural ones could also be related to the background of the participants. On the other hand, because these professionals collaborate closely with colleagues working on the infrastructural side, they still provided many insights into the role of infrastructural instruments and their potential across fields and contexts.

## Discussion

### Discussion of instrument framework and new instruments identified

The initial, conceptual framework created based on the literature review in this study was refined based on the interview findings. Compared to the conceptual framework, the refined, empirically-enhanced framework developed in this study provides more specific insights into the level of influence of the identified generic infrastructural and institutional instruments for openly sharing and reusing research data in a specific research field. For example, it shows that some instruments have a very high influence on data sharing and reuse, whereas others barely have any influence. While this refined framework is the first of its kind, in parallel, various other projects are being conducted in which elements of frameworks for data sharing and reuse in Epidemiology are being developed. For instance, one of the key objectives of the German NFDI4Health initiative includes utilizing a framework to enable single-access to health data from decentralized Epidemiological data infrastructures [83]. While our framework stimulates a more theoretical discussion on what institutional and infrastructural instruments should be prioritized in Epidemiology, the NFDI4Health framework offers services to increase the findability, accessibility, interoperability and reusability (or FAIRness) of health data in practice, such as publication guidelines, a metadata schema and a study portal. Several other studies have also explored data sharing in Epidemiology, such as [84,85], but they do not examine the level and type of influence of infrastructural and institutional arrangements on open research data sharing and reuse practices.

Moreover, the refined framework revealed several new instruments influencing open research data sharing and reuse in Epidemiology that were identified through the interviews. Compared to the literature, the interviews led to the identification of three new infrastructural instruments (1. a fast download process, 2. a standardized way of working among different repositories, 3. the enhanced usage of unique identifiers) and two new institutional instruments (1. building an open science community within the Epidemiology field, 2. increased communication among the scientific community so that two people with similar research interests are aware of each other). Note that these instruments were each mentioned only by one or two interviewees. The type and amount of influence they have on open research data sharing and reuse requires further investigation.

### Characterizing the field of Epidemiology

To contextualize and interpret the results of this study, it is important to highlight a number of Epidemiology-specific characteristics. First, Clinical Epidemiologists in general have a medical degree and professional training, provide patient care/treatment in addition to conducting research and teaching [I3]. This is relevant for our study as clinical work has consequences in terms of time use and time resources that can be allocated for data sharing practices. Moreover, clinical work is usually under time pressure: the healthcare sector in the Netherlands has been dealing with shortages of medical personnel for many years [86].

Second, the fields of public health and other fields in Epidemiology are strongly bounded by the General Data Protection Regulation (GDPR) in (open) data sharing because of the amount of personal and highly sensitive data with which they work. For example, all participants who contributed data to a cohort have the right to be informed which research project is using their personal data and to reject the use of their data for specific projects (i.e., dynamic consent). Meanwhile, the full anonymization of datasets in Epidemiology lowers the scientific value of data significantly. Data linkages are important in maximizing the value of datasets in this field, but linking datasets when they are fully anonymized is a challenge. Related to this, since Epidemiology researchers often work with data on human subjects, they need to obtain approval from Institutional Review Boards or Human Research Ethics Committees before they are allowed to openly share their data. Thus, researchers working with human research data do not have full control over the decision of whether their research data will be shared openly or not. Institutional Review Boards or Human Research Ethics Committees play a key role in guaranteeing appropriate participant protections in scientific research [87]. In addition, in Epidemiology, trust centers are sometimes involved when it comes to sharing personal health data. Trust centers are technical and administrative units that anonymize or pseudonymize data before data sharing takes place. The work of such centers shows that data sharing is often only possible if data requesters are signing various legal documents enforcing special responsibilities. The importance of data protection in Epidemiology may be an important reason for our interviewees to perceive working with research data managers and offering support for legal aspects concerning open data practices as a highly important institutional instruments for openly sharing and reusing research data.

Third, for Epidemiology, understanding data collection methods is very important to researchers. In Epidemiological research, there is a strong relationship between a chosen data collection method, the associated error of measurement, and the associated research result [88]. As the choice of measurement tools significantly affects research results, Epidemiology researchers must always consider the impact of such choices during research [88]. Therefore, knowing exactly how a variable was measured (e.g., was the data self-measured by the primary researcher, or was it solely reported by the research participants?), is essential. This could be the reason why the interviewees in this study perceive metadata concerning data collection as highly important for open data sharing and reuse in Epidemiology.

Fourth, researchers in Epidemiology (and healthcare in general) need to be able to make advanced search queries on search engines like PubMed when searching for references. Because the field of healthcare collects patient level information on a continuous basis, there is a daily flow of new data (e.g., through new trials) flowing into the medical (research) databases, and there is a “sea of information” where researchers could easily get lost unless satisfactory search engines are used [89]. There is a separate line of literature supporting researchers with search strategies (see Fatehi, Gray [90] and Motschall and Falck-Ytter [91] for examples). The enormous data collection in Epidemiology may be one of the reasons why the interviewees emphasized the challenge of findings useful openly available research data, showing the importance of powerful search engines and easy-to-use interfaces in Epidemiology. Powerful search engines and easy-to-use interfaces were among the instruments that the interviews in this study perceived as highly important.

Fifth, Epidemiological cohort studies may take years, if not decades. The immense amount of effort that researchers have to put into collecting these datasets (both financial and time-wise efforts) enhances the beliefs of data ownership, compared to fields where data are collected from more centralized, automated sources, such as in the field of Astrophysics [92].

Finally, in Epidemiology, research agendas are likely to be flexible: researchers often develop their research questions during a data collection process. As Epidemiology examines the health-related states and events that affect populations, such as pandemic and epidemic-prone diseases, they are not always predictable such as in the case of COVID-19. Researchers race with time when these diseases happen, which means the data sharing has to happen faster compared to other fields where human engagement is lower. Data can lose relevance quickly. This also has implications for the level and nature of support (legal, ICT, etc.) that researchers need for research data management, which was also visible in the interviewees’ perceived importance of such support for open research data sharing and reuse in Epidemiology.

### Scientific implications

Previous research has extensively examined the benefits, barriers, motivators, and factors of (open) research data sharing and reuse [e.g., 1,2,9,66,93]. Some studies focus on open research data sharing and reuse in specific research fields, such as Geophysics [94] and Biomedicine [95], often in fields that generally have higher levels of open research data adoption. Previous research has not examined open research data adoption specifically in the context of Epidemiology, a field in which open data sharing and reuse are considered to be at lower levels. Considering this difference in data sharing levels, our study enables future research to make systematic comparisons between fields with varying data sharing levels to get insights into success and failure criteria of open research data adoption.

Furthermore, our conceptual framework could be a starting point for future research for studies in other research fields. For example, regarding infrastructural instruments, our conceptual framework delineates the functional and nonfunctional requirements for open data infrastructures to (theoretically) enhance data sharing and reuse. Because a (functional) data infrastructure is a necessity for open data sharing for any field, researchers who intend to study how open data adoption can be enhanced in other fields can use the same framework to establish why the level of data sharing in a certain field could be particularly high (or low), or to assess how well open data infrastructures perform (or fail to perform) in these specific fields, without the need for a new extensive literature review. For the field of Epidemiology in particular, our refined conceptual framework established could be a starting point for studies that will perform similar studies in Epidemiology focusing on different countries or subfields. Testing the refined conceptual framework in other contexts within Epidemiology such as other countries would help in understanding whether the instruments that are found to have (medium, high, or very high) influence in our study are also valued elsewhere, with the intention to establish the generalizability of study’s results.

Moreover, despite a few exceptions (e.g., [35]), there is barely any literature addressing the topic of infrastructural and institutional instruments to promote openly sharing and reusing open research data. [35] assess how infrastructural and institutional instruments can be used to enhance open research data adoption, but this study concentrates on a specific university rather than a specific research field as we did in this study. Therefore, the novelty of our study comes from examining open research data sharing and reuse practices (1) using this novel concept of infrastructural and institutional instruments, and at the same time (2) performing this study in a specific research discipline. This study is, to our best knowledge, the first study that focused on the Epidemiology field while examining the roles of instruments based on field-dependent characteristics in relation to open research data.

### Societal implications

This study examines ways of addressing the barriers with open research data sharing and reuse are faced. Tackling the barriers to open research data adoption benefits society in various ways. First, open research data can benefit researchers, because researchers who share their research data along with their publications could increase their impact, through for example more citations for the associated research article as well as citations for the research data itself [7]. Moreover, if a researcher openly shares a dataset that was used for a journal article, others could replicate the results, confirming the validity of the original research results. Reusing openly shared data saves time and effort in data collection processes [2]. Considering that Epidemiological studies can be extremely costly, this is highly valuable. Researchers can combine various datasets from different sources and perform meta-analyses to produce novel research findings [10].

Besides potential benefits at the individual researcher level, there are also various benefits for science and scientific disciplines in general. For instance, enhanced data sharing potentially benefits scientific research by preventing research misconduct (e.g., the fabrication and falsification of research data), reducing errors in research results, and building transparency to research processes [2,7]. As making research datasets openly available increases the visibility of the researcher and research outputs, collaborations in the scientific field can also increase [10].

The field of Epidemiology specifically would benefit from increased open research data sharing to help understand diseases faster as access to data is a vital prerequisite for identifying a public health problem that necessitates an urgent response [96]. For instance, during the Covid-19 pandemic, by openly sharing research data about the SARS-CoV-2 genome, researchers in China helped the researchers in other parts of the world to develop critical diagnostic methods and helped facilitate pandemic response activities [97].

Open research data adoption could also positively affect the public’s relationship with research and researchers. Because of increased transparency of the research processes and enhanced perception of scientific knowledge being a public good, the society would build more trust in research and show more willingness to attribute funding for research. Furthermore, open research data adoption can remove the financial barriers in front of research in low- and middle-income countries, and lower inequalities due to the imbalance of research resources across the globe [98].

Moreover, our approach of transforming behavioral elements (e.g., expectations and motivations of researchers) into tangible requirements for technical and organizational environments can guide stakeholders such as open data infrastructure developers, open data policy makers at universities and funding agencies, university libraries, lawmakers, and governmental policymakers, who aim at effective interventions that lead to increased levels of open data sharing and reuse in practice. Since our study gives insights into the usability of open data infrastructures, infrastructure developers can use these insights when operationalizing usability in infrastructure design. Our study can inform governmental policymakers and lawmakers who want to tackle the barriers to data sharing stemming from the GDPR. Given that there are many possible strategies that can be used to increase open data sharing, university policymakers can prioritize their interventions based on our study’s indications of which of these tools are more promising than others. Our study provides policymakers with a better understanding of what type of interventions could result in increased open data sharing practices (or what kind of interventions may rather be ineffective). For example, our study indicated that simply pushing researchers to share research data in the form of (coercive) policies may be an ineffective method. Our study can help librarians to understand how they can effectively broaden their roles in research data management support and in which ways they should increase their capabilities, considering the types of support researchers (will) need in the long run. Finally, our study can show university legal teams what to consider when shaping their communications with researchers in the context of open data practices.

### Research limitations and directions for future research

This section discusses this study’s main limitations and relate those to various avenues for future research. First, despite the fact that interviewees from as many University Medical Centers in the Netherlands as possible participated in this study, with different backgrounds and contexts (e.g., different universities) to ensure a variety of perspectives on the topic under investigation, it is not possible to make statements at the level of specific types/levels of Epidemiology researchers (e.g., PhD candidates or professors) due to the different contexts in which they operate. In addition, our findings concerning the identified infrastructural and institutional instruments are derived from a study with ten Epidemiology researchers and one data steward. It is not known whether the ten researchers and the single data steward are representative of the Epidemiology discipline as only seven sub-fields in Epidemiology, including Environmental Epidemiology, General Epidemiology, and Clinical Epidemiology were covered. Data sharing practices may differ within each of those sub fields, and thus this requires further analysis. Open research data in Clinical and Genetic Epidemiology, for example, may raise specific issues compared to other areas in Epidemiology such as clinical or genetic data derived from cancer registries. These data cannot be shared openly on the internet. For such types of data, there may be more restricted levels of openness or alternative ways of data analysis.

Furthermore, our research led to a list of desired or ideal infrastructural and institutional instruments. In the prioritization exercise to determine the availability and importance of the infrastructural and institutional instruments based on the interview results, we used the responses with a definitive answer and not the ones about which respondents were unsure. It can be argued that, on top of the responses that included a concrete (definitive) answer (i.e., a solid ‘important’ or ‘not important’), the omitted responses may also have influenced the analysis and results, for example if the omission is due to an interviewee being “unsure” about the answer. Sometimes, the interviewees understood the question but was not so “sure” of the answer or was not familiar with the instrument that was mentioned. The omission of the non-definitive responses may have affected the calculations. Moreover, implementing the prioritized infrastructural and institutional instruments in practice requires substantial resources, including financial investments and capacity. The financial implications of implementing just the most important instruments in our ranking are significant.

Other limitations of this study relate to its qualitative nature and the analysis of the collected qualitative data. Our qualitative research approach enabled us to obtain in-depth information and insights. A drawback is that researchers may have provided socially desirable or otherwise biased responses to our behavioral questions in the interviews, that may have affected the study results’ validity. Furthermore, although a systematic approach was used to operationalize the collected data, there is always a chance that the data analysis procedure is biased despite efforts to minimize the risk of biased data analysis by asking interviewees to review our interview summaries before analyzing them, by investigating secondary data sources (i.e. policy documents), by triangulating data collection methods (i.e., interviews and a research workshop), and by collecting feedback on the research approach and findings from experts in qualitative research methods and open data research.

Our findings provide a basis for future research by open data scholars to analyze the qualitative aspects of our data collection beyond the confines of the conceptual framework or the specific scope of the study, and to replicate our study involving more Epidemiology researchers, to investigate whether the findings of this study can be validated and generalized to the wider population of Epidemiology researchers. Involving other actors than researchers, such as policymakers and librarians at universities or developers of open data infrastructure at the national level would further insights on how the identified infrastructural and institutional arrangements can be operationalized in practice. Moreover, further research is needed to explore whether the identified infrastructural and institutional instruments could indeed improve open data sharing and reuse in other fields than epidemiology, as suggested by the workshop participants.

## Conclusion

This study addresses two research questions: 1) What influence do infrastructural and institutional arrangements have on open research data sharing and reuse practices in the field of Epidemiology? And 2) how could infrastructural and institutional instruments used in Epidemiology potentially be useful to other research disciplines? The six infrastructural instruments that our interviewees refer to as being highly important for open data sharing and reuse in Epidemiology concern: 1) easy-to-use interfaces, 2) compatibility between different data infrastructures, 3) the availability of powerful search engines, 4) the availability of an overarching registry of repositories, 5) infrastructures providing metadata concerning data collection, and 6) the compatibility of the infrastructure with domain-specific privacy needs. Furthermore, our interviewees perceive four institutional instruments as being highly important for openly sharing and reusing research data in Epidemiology: 1) data steward support, 2) working with research data managers, 3) offering support for legal aspects concerning open data practices, and 4) recognizing and rewarding the sharing of open research data sharing. As far as the second research question is concerned, our findings show that many of the challenges faced in Epidemiology are common in other research fields. In addition, our conceptual framework reveals a number of challenges and related solutions or facilitators that are specific to Epidemiology, as well as the potential for open data infrastructure developers, policymakers, and research funding organizations to develop field-independent institutional and infrastructural instruments to stimulate open research data. This study complements scientists’ understanding of field-specific facilitators and challenges for open research data.

## Supporting information

S1 File Interview questions. Underlying research data–The codebook underlying this study is openly available through the 4TU.ResearchData repository at http://doi.org/10.4121/20085560.
(DOCX)
