# Supplementary material for: Identifying the most important facilitators of open research data sharing and reuse in Epidemiology: A mixed-methods study
Source: PLoS One. 2024 Feb 8;19(2):e0297969. doi: 10.1371/journal.pone.0297969 (PMC10852342; doi:10.1371/journal.pone.0297969)
Supplement: S1 File — Underlying research data–The codebook underlying this study is openly available through the 4TU.ResearchData repository at http://doi.org/10.4121/20085560. (DOCX) [file pone.0297969.s001.docx]

## Appendix A: Interview design (for researchers)

**Research Data Sharing and Reuse: a Case Study in Epidemiology – Interview Questions**

**Interview information**

Name interviewer:

Interview number:

Interview date:

Title and name respondent:

Organization where the respondent works:

Country where the respondent works: Netherlands

**Introduction**

**Welcome**

Welcome, and thank you for participating in this research. My name is <name>. I conduct this research as part of <…> project, which is a case study in the field of Epidemiology on open research data sharing and reuse.

**Research objective**

In this study, we examine the infrastructural and institutional instruments that are used in the field of Epidemiology to stimulate openly sharing and reusing research data. This study’s objective is to understand what role infrastructural and institutional arrangements can play in promoting open data sharing and use behaviour in Epidemiology.

I will ask you about 16 questions in five categories.

The interview takes about 1 hour.

I will share my notes with you for you to revise and approve before including them in my thesis.

**After the interview**

When I conclude my <…> project in July or August 2022, I will share my project report with you.

**Interview questions**

This interview consists of five sections, namely:

1. Background information

2. Your involvement in open research data, open research data sharing, and reuse

3. Infrastructural instruments that influence your motivation and behaviour towards openly sharing and re-using research data

4. Institutional instruments that influence your motivation and behaviour towards openly sharing and re-using research data

5. Barriers to open research data sharing and reuse

**Section 1: Background information**

In this section, you are asked to provide information about your background. This data is personal and we will only report on this anonymously.

1. What is your age?
2. What is your current position in your institution?

- Ph.D. candidate
- Postdoctoral researcher
- Assistant professor
- Associate professor
- Full professor
- Other (please specify) ____________________________________________________

1. For how long (i.e. years) have you been in this academic/scientific position?
2. In which subfield of Epidemiology are you currently employed?

**Section 2: Experience with open research data sharing and reuse**

My research focuses on open research data. When I refer to open research data, I mean data that is structured, machine-readable data, actively published on the internet. Open data is published for public reuse, and is ideally also Findable, Accessible, Interoperable, and Reusable (FAIR). Open data can be both quantitative and qualitative and can be either raw/primary, derived from primary data for subsequent analysis or interpretation, or derived from existing sources.

1. Do you have any experience with openly sharing research data? Could you provide more detail?
2. Do you have any experience with reusing research data that others have openly shared? Could you provide more detail?

**Section 3: Infrastructural instruments for openly sharing and re-using research data**

In this study, we examine the infrastructural and institutional instruments that are used to stimulate openly sharing and re-using open research data.

I would now like to discuss the infrastructural instruments with you, to understand to what extent these instruments affect your research data sharing and reuse behaviour.

In my literature review on infrastructural instruments, I have identified various infrastructural instruments that may affect open data sharing and reuse motivation and behaviour. Please note that when I refer to ‘(open data) infrastructures’ in the following questions, I mean (technical) infrastructures that you can use when you are engaging with open data sharing and reuse activities. Such infrastructures could be data repositories; software and tools that can be used for finding, storing, curating data, metadata creation, anonymization, analysis, research data management, licensing, etc.

1. Could you indicate, for each of the following instruments, whether you have access to these instruments and explain to what extent they affect your open research data sharing and reuse activities? Please answer the questions according to the infrastructures that you use.

| **Infrastructural instruments** | **Do you agree with the statement?**  **(yes, no, partially)**  **Please explain why.** | **To what extent does this instrument influence your open research data sharing and reuse behaviour?** |
| --- | --- | --- |
| The open data infrastructure(s) that I use has (have) user-friendly graphic interfaces. |  |  |
| The open data infrastructure(s) that I use allows (allow) for easy and quick data analysis. |  |  |
| The open data infrastructure(s) that I use is (are) easy to use. |  |  |
| The open data infrastructure(s) that I use is (are) compatible with the different types of data that are used in my field. |  |  |
| The open data infrastructure(s) that I use is (are) reliable. |  |  |
| The open data infrastructure(s) that I use can accommodate a large volume of data. |  |  |
| The open data infrastructure(s) that I use helps (help) with choosing a license (e.g. CC0, CC-BY., etc.). |  |  |
| The infrastructures (e.g. data repositories; software that are used for metadata creation, anonymization, etc.) that I use are integrated with each other and compatible. |  |  |
| The search engine(s) on the open data repository that I use is (are) sufficient for my open data search needs. |  |  |
| The open data infrastructure(s) that I use helps (help) with the selection of an appropriate repository for openly sharing research data. |  |  |
| A data management tool is offered to me. |  |  |
| The open data repository that I use accommodates metadata standards (i.e. enabling to properly store metadata and to view metadata of other datasets) |  |  |
| The open data repository that I use helps with the creation of an appropriate citation for the data. |  |  |
| Tools for metadata creation and management are offered to me. |  |  |
| The open data infrastructure(s) that I use incentivizes (incentivize) usage of metadata standards (e.g. by explicitly asking for the usage of standards when sharing your research data). |  |  |
| The open data repository that I use is linked to overarching/ aggregating infrastructures (i.e. registry of repositories) which help searching for data across different data repositories. |  |  |
| The open data repository that I use requires the data depositor to provide metadata on the data collection methods. |  |  |
| The open data infrastructure(s) that I use presents (present) data usage statistics. |  |  |
| The open data infrastructure(s) that I use is (are) trustworthy (e.g. in terms of securely storing data, against breach). |  |  |
| Ways/methods to assess how trustworthy an open data repository or an open data set are offered to me. |  |  |
| Tools for data anonymization are offered to me. |  |  |
| On the open data repository that I use, there are different access restriction types to choose from (i.e. giving ability to place conditions on data access). |  |  |

1. Are there any other existing infrastructural instruments that make it easier for you to participate in open data sharing and reuse, or that incentivize or facilitate open data sharing and reuse?
2. Can you think of any other functionalities that you wish open data infrastructures had, so that you would be more stimulated towards openly sharing and reusing research data? (Alternatively, you can indicate the troublesome features about the infrastructures that you wish would be fixed)
3. Which infrastructural instruments do you believe stimulate openly sharing research data and reusing openly shared research data the most in your field?

**Section 4: Institutional instruments for openly sharing and re-using research data**

I would now like to discuss institutional instruments for open research data. When I refer to *institutional instruments,* I refer to the combination of formal structures (e.g., policy, processes), informal structures (e.g. norms, culture), and more enforcing or operational mechanisms that institutions can implement to stimulate openly sharing research data and reusing open research data.

1. Please answer the questions according to the institutional context under which you conduct research and engage in open research data sharing and reuse activities.

| **Institutional instruments** | **Do you agree with the statement?**  **(yes, no, partially)**  **Please explain why.** | **To what extent does this instrument influence your open research data sharing and reuse behaviour?** |
| --- | --- | --- |
| There is an institutional data sharing policy and/or there are guidelines for openly sharing or reusing research data in my organization. |  |  |
| There is an institutional data management policy and/or a data deletion policy and/or data security policy in my organization. |  |  |
| My organisation requires me to create a data management plan (DMP) as a necessary part of the research cycle. (i.e. asking the researcher to think about costs related to access, management, and preservation of data before the research starts) |  |  |
| My organisation provides support for understanding and fulfilling legal requirements (“legal basis for rights of use”) regarding openly sharing or reusing research data. |  |  |
| Different data management policies and guidelines (that I am aware of) are aligned and consistent with one another. |  |  |
| My organisation provides guidelines on obtaining consent for (open) data sharing. |  |  |
| My organisation provides guidelines on data anonymization. |  |  |
| My organization’s library provides support regarding non-technical topics such as choosing appropriate open data tools, selection of repositories; and/or regarding technical support such as digital curation of data, preparing datasets for a repository, accessing a repository, archiving data, backup practices, removing data from repositories, and creating metadata for datasets. |  |  |
| My organization’s legal departments provide support regarding open research data sharing and reuse. (Such support could be in terms of privacy, data ownership, copyright, etc.) |  |  |
| My organization’s data stewards provide support. (e.g., possibility to ask questions to the data steward about open research data sharing and reuse) |  |  |
| I have the ability to hire data managers to take care of my research data management activities. |  |  |
| My organization’s website gives information and guidance on data management and open data sharing and reuse requirements. (e.g. researcher can easily reach relevant guidelines via organization’s websites) |  |  |
| My organization provides training and educational support (seminars, courses, training modules, etc.), for instance, on topics of open science, data management, technical training on archiving/backup, digital description or curation of data sets, data anonymization, etc. |  |  |
| My organization or my field offers financial resources such as separate funds for treatment and management of openly shared research data. |  |  |
| (open) data sharing is framed as a concrete goal in my organization and my organization’s policy (documents). |  |  |
| There is a data-sharing culture in my organization. |  |  |
| Open research data contributions are recognized and rewarded in my organization and/or in my field. |  |  |
| Data sharing contributions are considered during hiring, tenure, and/or promotion decisions in my organization or my field. |  |  |
| My organization and/or my field uses track metrics for data sharing contributions. |  |  |
| There is a data citation policy in my organization. |  |  |
| The academic journals in my field mandate or request me to openly share research data. |  |  |
| The funders in my field mandate or request me to openly share research data. |  |  |
| My organization mandates or requests me to openly share research data. |  |  |
| My organization publishes experiences in research data sharing on its website to promote open data. |  |  |
| My organization helps me to comprehend the benefits of data sharing and the needs for data sharing. |  |  |
| My organization helps me to understand ways to tackle issues around data ownership, ethics, and privacy in open data. |  |  |

1. Are there any other existing institutional instruments that facilitate or incentivize open data sharing and reuse? (You can think of such instruments by considering what kind of formal (policies, rules), informal structures (norm, culture), or enforcement mechanisms are used in your organization that incentivize, ease or facilitate your open research data sharing and reusing behaviour.)
2. Apart from the topics we discussed, what kind of support do you wish to receive from your organisation so that you would be more stimulated towards openly sharing and reusing research data? (Alternatively, you can indicate the troublesome institutional/organizational issues that that you wish would be solved)
3. Which institutional instruments do you believe stimulate openly sharing research data and reusing openly shared research data the most in your field?

**Part 5: Barriers to open research data sharing and reuse**

1. Despite the discussed instruments, which factors inhibit *openly sharing* research data on a large scale in your field?
2. Despite the discussed instruments, which factors inhibit the *reuse* of open research data on a large scale in your field?

**Finalizing the interview**

This is the end of this interview. Thank you very much for your cooperation. I will process this interview and send you a summary of your answers to the questions so that you will be able to review your answers and/or add anything you wish.

Do you have any final questions or additions concerning this interview?

…………………….

Then I wish you a nice day and again thank you so much for your participation in my research.

## Appendix B: Interview design (for the research data management consultant)

**Research Data Sharing and Reuse: a Case Study in Epidemiology – Interview Questions**

**Introduction**

**Research objective**

In this study, we examine the infrastructural and institutional instruments that are used in the field of Epidemiology to stimulate openly sharing and reusing research data. This study’s objective is to understand what role infrastructural and institutional arrangements can play in promoting open data sharing and use behaviour in Epidemiology. So far, my primary source of information has been epidemiology researchers.

During the interviews I had with Epidemiology researchers, it was brought up by several researchers that the legal context forming the boundary of data sharing in Epidemiology influences open data practices in the field heavily. It was mentioned several times that open research data for the field of human health is usually not fully guided by individual researchers’ behaviour, but rather it is significantly bounded by GDPR privacy laws and informed consent procedures. Therefore, in light of these developments I wanted to interview somebody who has expertise on data protection issues to fully understand how the issues stemming from privacy regulations are, and to what extent these can be tackled using infrastructural and institutional instruments.

I will ask you about 20 questions in 4 categories.

The interview takes roughly 40 mins

I will share my notes with you for you to revise and approve before including them in my thesis.

**After the interview**

When I conclude my master thesis project in July or August this year, I will share my master thesis project report with you.

**Interview questions**

This interview consists of five sections, namely:

1. Background information

2. Open research data sharing and reuse in the field of Epidemiology

3. GDPR and informed consent as barriers to open research data sharing

4. Infrastructural and institutional instruments that are used to stimulate openly sharing and reusing research data

**Section 1: Background information**

In this section, you are asked to provide information about your background. This data is personal and we will only report on this anonymously.

1. What is your current position in your institution?
2. Do you have a specific research theme (field) that you are engaging with as part of your current position?

**Section 2: Open research data sharing and reuse in the field of Epidemiology**

My research focuses on open research data. When I refer to open research data, I mean data that is structured, actively published on the internet. Open data is published for public reuse, and is ideally also Findable, Accessible, Interoperable, and Reusable (FAIR). Open data can be both quantitative and qualitative and can be either raw/primary, derived from primary data for subsequent analysis or interpretation, or derived from existing sources.

1. How does your current position relate to open research data sharing in epidemiology? How do you engage/work with epidemiology researchers regarding open research data sharing?
2. Can you talk about the characteristics of open data sharing practices in the field of epidemiology?

*-Which repositories exist for epidemiology?*

*-What type of data types are being openly shared in the field of epidemiology? What are the characteristics of the data that are being shared openly?*

*-What is, in your opinion, the level of open research data sharing in the field?*

*-How much influence do researchers actually have on open data sharing or reuse?*

*-Which types of data sharing are more prevalent in the field? (open data sharing, data sharing by collaboration, data sharing by request, etc)*

*-How do data sharing practices differ from other close or far research fields? Does the level of data sharing in epidemiology differ with respect to other research fields?*

1. What are the biggest barriers to open data sharing and reuse activities in the field of epidemiology? What are the biggest struggles for researchers in your opinion? What demotivates them?

**Section 3: GDPR and informed consent as barriers to open research data sharing**

1. What are the current GDPR and informed consent regulations that affect open research data sharing and reuse practices in the field of Epidemiology? What do researchers have to do in order to comply with the requirements if they would like to openly share research data or reuse openly shared research data?
2. How do GDPR and informed consent requirements affect (the level of) open research data sharing in the field of epidemiology? Do you believe that these regulations and requirements are important barriers to open data practices?
3. Are there any other regulations/requirements that affect (or come as a barrier to) open research data sharing and reuse practices in the field of epidemiology?
4. Do you have any ideas on how the barriers to open data practices due to GDPR and informed consent regulations could be solved?
5. Apart from sharing primary/raw data, what are the possibilities of making metadata or summary statistics openly available in the field of epidemiology? Do GDPR and informed consent regulations also pose a problem to sharing such types of data?

**Section 4: Infrastructural and institutional instruments that are used to stimulate openly sharing and reusing research data**

1. Does your organization have any institutional (open) data sharing policies or policies for research data management? How do these policies affect open data sharing and reuse practices?
2. Do you think your organization provides sufficient support for explaining the legal requirements of open data practices to researchers and helping them comply with such requirements?
3. How does your organization try to promote/facilitate open data sharing and reuse practices? Do you think these promotion activities are sufficient for the field of epidemiology?
4. Do you think offering more financial resources such as separate funds for treatment and management of openly shared research data could be a positive influence for open data practices in the field of epidemiology?
5. What kind of support do you think researchers are expecting to receive from your organisation so that they would be more stimulated towards openly sharing and reusing research data? What are the troublesome organizational issues with which the researchers are struggling regarding open data practices?
6. Does your institution have any data infrastructure (such as an open data repository, or a specific data (management) software) that has been built or adopted to promote open data practices?
7. To what extent would data anonymization address the issues on open research data sharing in the field of epidemiology? What are the roles, functions, benefits of data anonymization tools in this regard?
8. Do you think the ease of use of data repositories is important for open data practices?
9. Do you think the availability of a search engine that satisfies open data search needs is important for open data practices?
10. Are there any functionalities/features that researchers wish to see in the open data infrastructures (such as the open data repositories), so that they would be more stimulated towards openly sharing and reusing research data? What are the troublesome features of open data infrastructures with which the researchers are struggling regarding open data practices?
